# Increased muscle force does not induce greater stretch-induced damage to calf muscles during work-matched heel drop exercise

**DOI:** 10.1007/s00421-023-05188-2

**Published:** 2023-04-13

**Authors:** Patricio A. Pincheira, Dean L. Mayfield, Aaron S. Fox, Nicholas A. T. Brown, Timothy J. Carroll, Andrew G. Cresswell, Glen A. Lichtwark

**Affiliations:** 1 School of Human Movement and Nutrition Sciences, The University of Queensland, Blair Drive 26B, St Lucia, Brisbane, 4067 Australia; 2 School of Health and Rehabilitation Sciences, The University of Queensland, Brisbane, Australia; 3 Department of Evolution, Ecology, and Organismal Biology, University of California, Riverside, USA; 4 Centre for Sport Research, School of Exercise and Nutrition Sciences, Deakin University, Geelong, Australia; 5 Faculty of Health, University of Canberra, Canberra, Australia

**Keywords:** Gastrocnemius muscle, Eccentric exercise, Ultrasonography, Surface electromyography, Exercise-induced muscle damage

## Abstract

**Purpose:**

To investigate the effect of muscle force during active stretch on quantitative and qualitative indicators of exercise-induced muscle damage (EIMD) in the medial gastrocnemius (MG) muscle.

**Methods:**

Twelve recreationally active volunteers performed two trials of an eccentric heel drop exercise. Participants performed a single bout of low-load (body weight) and high-load (body weight + 30% body weight) exercises on separate legs. The total mechanical work output for each condition was matched between legs. Before, two hours and 48 h after each bout of eccentric exercise, electrically stimulated triceps surae twitch torque, muscle soreness, MG active fascicle length at maximum twitch torque and muscle passive stiffness were collected. Triceps surae electromyographic (EMG) activity, MG fascicle stretch and MG muscle–tendon unit (MTU) length were measured during the eccentric tasks.

**Results:**

The high-load condition increased triceps surae muscle activity by 6–9%, but reduced MG fascicle stretch (*p* < 0.001). MTU stretch was similar between conditions. The greater muscle force during stretch did not give rise to additional torque loss (5 vs 6%) or intensify muscle soreness.

**Conclusions:**

Adding 30% body weight during eccentric contractions has a modest impact on exercise-induced muscle damage in the medial gastrocnemius muscle. These results suggest that muscle load may not be an important determinant of stretch-induced muscle damage in the human MG muscle. The muscle investigated does exhibit large pennation angles and high series elastic compliance; architectural features that likely buffer muscle fibres against stretch and damage.

## Introduction

Exercise-induced muscle damage (EIMD) frequently occurs after unaccustomed exercise. EIMD is usually associated with myofibrillar disturbances, Z-line streaming and impairment in excitation–contraction coupling (Clarkson and Hubal 2002; Hyldahl and Hubal 2014). These impairments often lead to prolonged force loss, muscle soreness and inflammation (Clarkson and Hubal 2002; Hyldahl and Hubal 2014). Due to the detrimental effects of EIMD on muscle performance, a thorough understanding of the factors that determine and mitigate its severity is critical to athletes preparing for competition as well as undergoing rehabilitation.

Eccentric contractions are often associated with high forces (i.e., loads) imposed on the muscle that can result in muscle damage (Warren et al. 1993; Lieber 2018). However, while some studies suggested that the magnitude of muscle force exerted during lengthening is the primary factor that regulates muscle injury magnitude (Warren et al. 1993; Black and McCully 2008), others did not (Lieber and Fridén 1993). It may be that differences in the mechanical work absorbed by the muscle (i.e., cumulative force applied to the muscle during a given lengthening) is the cause of these discrepancies (Brooks and Faulkner 1996). The relationship between force and muscle damage when controlling for total muscle work remains uncertain and should be investigated.

While muscle soreness can be experienced after repeated eccentric contractions in the lower limbs (Peñailillo et al. 2015; Guilhem et al. 2016; Pincheira et al. 2018; Chen et al. 2019), the loss of force is often much less than that which is reported in muscles of the upper limb for comparable work rates and stretch amplitudes (Chen et al. 2019). One potential reason for reduced contractile damage in lower limb muscles is that fibre stretch is buffered by stretch of the series elastic element of the muscle (e.g., Achilles tendon) (Griffiths 1991; Hoffman et al. 2014). Indeed, during a controlled eccentric contraction, the gastrocnemius fascicles undergo only ~ 17% of the stretch experienced by the entire muscle–tendon unit (Pincheira et al. 2018). Tendon stretch increases with applied muscle force. Hence, we predict that the absolute length of fascicles may be shorter during eccentric contractions at higher forces when the length trajectory of the muscle–tendon unit (MTU) is similar. Because active muscles operating at long lengths are usually associated with higher levels of muscle damage (Talbot and Morgan 1998; Guilhem et al. 2016), active muscles operating at higher forces and at shorter lengths, may non-intuitively, undergo lower levels of muscle damage in comparison to muscles operating at lower forces at longer lengths. Hence, the trade-off between force and operating length of muscle fibres in lower limb muscles requires further examination.

This study aimed to assess the effect of muscle force during active lengthening contractions on quantitative and qualitative indicators of EIMD in the medial gastrocnemius (MG) muscle. In an ecologically valid in vivo study design, we manipulated muscle force by changing the load (body weight (BW)) experienced by the participant during unipedal heel drop exercises, while controlling for total muscle mechanical work (negative). We hypothesised that high muscle load requirements would not substantially increase the indicators of EIMD in the MG, due to lower absolute stretch of fibres in comparison to lower force requirements.

## Methods

Twelve recreationally-active volunteers (age 21 ± 2.8 years, mass 71.9 ± 13.5 kg, height 178.2 ± 11.1 cm; six females) provided written informed consent to participate in the study. Participants were excluded if they had any pre-existing lower limb injuries in the past six months and/or had undertaken eccentric training exercises specific to the calf muscle group within six months before the experiment. The protocol was approved by the local university ethics committee and conducted according to the Declaration of Helsinki.

The exercise consisted of single-leg eccentric heel drops performed on the edge of a wooden step as previously described (Alfredson et al. 1998). In brief, standing upright with the knee fully extended, BW on the forefoot, and the ankle joint in full plantar flexion, the ankle extensors were eccentrically loaded by having the participant lower the heel beneath the level of the forefoot. The non-test leg returned the participant to the start position such that the test leg performed only eccentric contractions. A metronome controlled exercise speed and frequency, while video feedback ensured that the ankle range of motion was consistent.

In a randomised within-subject design, participants performed one bout of eccentric exercise with each leg as part of either a low- or high-load condition. The low-load condition consisted of heel drops performed with the participant’s BW. The high-load condition consisted of performing heels drops with BW while wearing a weight vest with 30% BW. This method of load standardisation has been used previously to set relative training/exercise loads for plantar flexor muscles in basic science/cross sectional studies (Pincheira et al. 2021a) and clinical/interventional (Alfredson et al. 1998; Mafi et al. 2001) studies. This added BW was selected based on previous studies (Pincheira et al. 2021a) and allows to maximise the exercise load of the triceps surae in an ecologically sound manner, while avoiding the interference of weight-vest-induced fatigue (e.g., shoulder/neck muscles) on exercise performance and movement kinematics. Further, this high-load condition largely exceeds the heavy-load prescribed in common heel drop protocols used for calf muscle training (Alfredson et al. 1998), and is similar to the increase in plantar flexor torque seen when transitioning from walking to jogging (Schache et al. 2011). Each condition was completed approximately two weeks apart. The exercise in each condition consisted of sets of ~ 20 heel drops separated by a 2-min rest period. The total work performed for each condition was matched between legs to standardise the training volume (see below for details).

To analyse the effect of load on MG EIMD, total twitch torque, MG soreness scores, MG passive stiffness and MG active fascicle length at maximum twitch torque were measured before (PRE), two hours (2H) and 48 h (48HR) after each bout of exercise. A significant change in total twitch torque and/or MG soreness was considered as evidence of EIMD (Hoffman et al. 2014; Pincheira et al. 2018). Twitch torques elicited by supramaximal electrical stimulation were used to assess force loss from the contractile tissue, avoiding any influence of peripheral muscle fatigue during post-exercise testing (Guilhem et al. 2016; Carroll et al. 2016). MG fascicle length changes during plantar flexion twitches and changes in passive stiffness were collected as complimentary muscle damage outcomes. MG stretch, triceps surae electromyographic (EMG) activity (MG, lateral gastrocnemius (LG) and soleus (SOL)), MG MTU stretch and ankle range of motion were collected to characterize lower leg neuromechanical behaviour during the exercise bouts.

Total twitch torque was determined using a modified version of a method (presented previously by our group) used to estimate the maximum torque occurring on the length-tension relationship of the gastrocnemius muscle (Hoffman et al. 2012, 2014; Pincheira et al. 2018, 2021a). Total twitch torque was calculated as the mean of peak plantar flexion twitch torques obtained across the ankle range of motion (15° plantar flexion to maximal dorsiflexion). Plantar flexion twitch torques were electrically-evoked by stimulating the tibial nerve percutaneously at the popliteal fossa with a constant-current stimulus (three square wave pulses, 20 ms inter-stimulus interval, 500 μs pulse width; DS7AH, Digitimer, Welwyn Garden City, UK). In brief, with the participants lying in the prone position and their foot firmly attached to a dynamometer footplate (Humac Norm, CSMi, Stoughton, USA), supramaximal stimuli (120% of the maximal twitch torque achieved at 15° of dorsiflexion) were applied at several different joint angles across the ankle range of motion (7 to 11 joint angles depending on the full range of motion of the participant) (Hoffman et al. 2014, 2016; Pincheira et al. 2018, 2021a). A single pulse was used 3–5 s prior to the triplet to minimize any thixotropic effect (Proske et al. 1993). Measurements were made for each twitch, at each joint angle, in a randomized fashion.

Self-reported muscle soreness was assessed using an algometer (Wagner Instruments, Riverside, USA). With the probe head of the algometer placed perpendicular to the mid-belly of the MG, the applied force was gradually increased until 2.5 kg of pressure was achieved. The participant then reported their perceived soreness of the muscle using a 10-point visual analogue scale (0: no soreness; 10: worst soreness ever felt).

MG passive stiffness was determined as the slope of a curve (*k*) fitted with an exponential expression to the relationship between passive MG fascicle length and passive plantar flexion torque (Hoffman et al. 2014, 2016; Pincheira et al. 2018). MG fascicle length was recorded during passive ankle rotations at a constant velocity (10 deg/s) through the full range of ankle motion, performed with the dynamometer described above. MG fascicles were captured using B-mode ultrasound (Echoblaster 128, LV7.5/60/96 40 mm transducer, 6 MHz, ~ 110 fps; Telemed, Vilnius, Lithuania). The flat-shaped transducer was firmly strapped to the MG using an elastic bandage. The location of the transducer relative to the skin was marked with an indelible marker for consistent placement in subsequent testing sessions. MG muscle fascicle length during passive ankle rotation was estimated offline using custom-written Matlab (The MathWorks, Natick, USA) scripts (Farris and Lichtwark 2016). Plantar flexion torque and ankle joint position were measured by the dynamometer and collected at a sampling rate of 2 kHz using an AD board (Micro 1401-3; Cambridge Electronic Design, Cambridge, UK).

MG fascicle length changes during plantar flexion twitches were measured using the ultrasonography methods described above. Ultrasound and torque measurements were synchronised via a continuous 5 V signal sent to the AD board from the ultrasound beamformer whenever fascicle images were being recorded. This method allowed active fascicle length to be quantified at the instant of peak plantar flexion torque during the elicited twitch. MG fascicle length was estimated automatically at the time of peak twitch torque utilising the Simple Muscle Architecture Analysis Software (Seynnes and Cronin 2020).

To analyse MG neuromechanical behaviour during the exercise bouts, lower limb kinematics, MG fascicle length and triceps surae muscle activation were measured synchronously. Lower limb kinematics were measured using an eight-camera optoelectronic motion capture system (200 Hz sampling frequency; Qualysis, Gothenburg, Sweden). Single reflective spherical markers were placed at anatomical landmarks on both legs as previously described (Hoffman et al. 2014, 2016). Labelled marker data were then exported to OpenSim, where a modified generic model (Arnold et al. 2010) scaled to each participant was used to estimate ankle range of motion (ROM) and MG MTU length in a process described elsewhere (Schache et al. 2013).

**Fig. 1:**
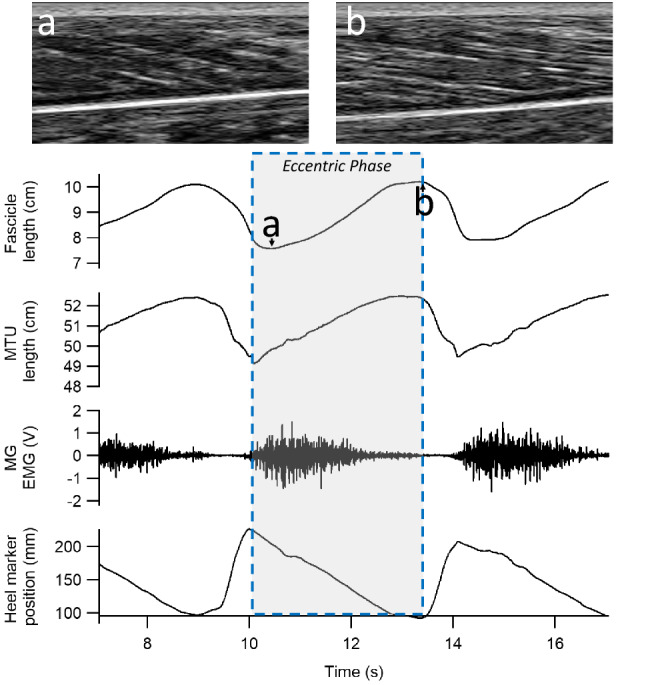
Neuromechanical measurements during the exercise bouts. Neuromechanical parameters of the medial gastrocnemius MG (fascicle length, muscle–tendon unit length (MTU), electromyography (EMG)) were estimated within the eccentric phase of the heel drops (shaded area within the blue dotted lines), defined by the vertical displacement of a marker placed on the heel. Ultrasound images of the change in fascicle lengths during one heel drop cycle are presented in panels (**a**) and (**b**)

MG fascicle length changes during the heel drops were recorded using the ultrasonography methods described above. The same custom-written Matlab scripts mentioned above (Farris and Lichtwark 2016) were used to track MG fascicle length during the exercise bouts. Ten cycles of heel drops (taken from the beginning and end of each exercise bout) were analysed. The MTU-level stretch phase of each cycle was determined using the 3D position of a reflective marker located on participant’s calcaneus (Fig. 1). Absolute stretch amplitude (i.e., difference between maximum and minimum length) was calculated within the stretch phase.

Surface EMG signals from LG, MG and SOL were also recorded during the heel drops. Bipolar electrodes (2 cm inter-electrode distance) were placed over the muscle bellies according to well-known anatomical guidelines (Hermens et al. 1999), after appropriate skin preparation (shaving and alcohol cleansing). EMG signals were sampled at a frequency of 2 kHz and bandwidth-filtered at 10–500 Hz (Neurolog NL900D, Digitimer, Hertforsdire, UK). For EMG normalisation purposes, in the same session that heel drops were performed, maximal voluntary isometric contractions (MVIC) were recorded. For MVIC recording, the participants laid in the prone position with their foot firmly strapped to the dynamometer mentioned above. Three MVICs (separated by 3 min of rest) were exerted with the ankle at neutral position (i.e., the sole of the foot perpendicular to the tibia). The highest MVIC value was used as a reference for EMG normalisation.

For EMG activity estimation, MG, LG and SOL EMG root mean square (RMS) activity (50 ms window) were calculated and averaged over the same 10 cycles where MG fascicle stretch was estimated. EMG signals were then normalised to the RMS EMG obtained during the MVICs. The RMS EMG normalised values were used for further analyses.

Before the statistical analysis, all variables were tested for normality using the Shapiro–Wilk test. Deviations from sphericity were corrected using the Greenhouse–Geisser method. To analyse the effect of load on the markers of muscle damage, two-way repeated-measures ANOVAs were performed to determine the effect of time (PRE, 2HR, 48HR) and load (low-load, high-load) on total twitch torque, soreness score, MG passive stiffness and active fascicle length at maximum twitch torque. Pre-planned post-hoc comparisons were made using Tukey’s test when a significant interaction or main effect was found. To analyse the effect of load on MG neuromechanical behaviour during the heel drops, paired t-tests were used to compare MG absolute stretch, MG MTU absolute stretch, ankle ROM and triceps surae EMG (MG, LG and SOL) between the high- and low-load conditions. Significant differences were established at *p* ≤ 0.05. The Šidák correction was applied for multiple comparisons. Data are reported as means and standard deviations. Effect sizes are reported as partial eta-squared (*η*_*p*_^2^).

## Results

The total work performed was similar between load conditions (low-load: 167.2 ± 29 J/kg; high-load 166.4 ± 28 J/kg). On average, 172 ± 18 and 96 ± 13 heel drops were performed in the low- and high-load conditions, respectively.

**Fig. 2:**
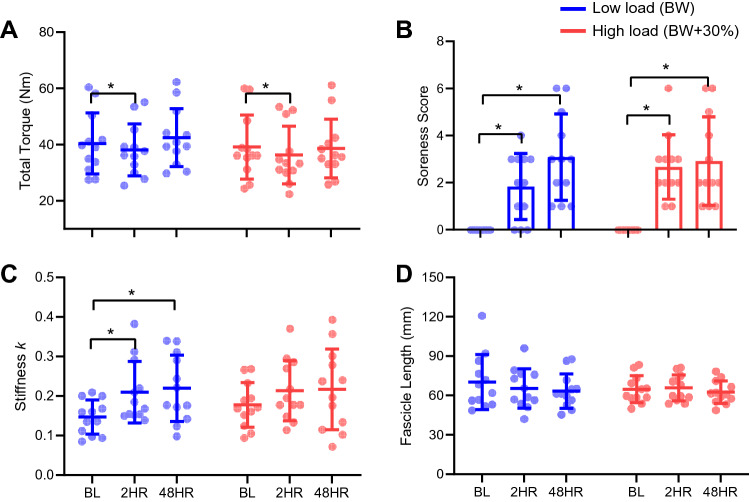
Medial gastrocnemius mechanical factors and soreness scores before and after eccentric exercise with different load levels. **A** total twitch torque; **B** soreness scores; **C** passive stiffness (*k*); **D** fascicle length. These variables were analyzed before (BL), two hours (2HR), and 48 h (48HR) after a bout of heel drops performed with a low (body weight) or high (body weight + 30% body weight) load condition in each leg. Individual values are presented as (circles), together with means and standard deviations (error bars). *Significant difference when comparing between time points (BL, 2HR, 48HR) (*p* < 0.05)

Changes in the main markers of muscle damage (i.e., total twitch torque and MG soreness scores) were evident following both low- and high-load conditions. However, no differences were found between load conditions. There was a main effect of time (*f*_2, 19_ = 15, *p* < 0.01, *η*_*p*_^2^ = 0.61) on total twitch torque, but no effect of load applied (f_1, 11_ = 2, *p* = 0.16, *η*_*p*_^2^ = 0.15) nor an interaction effect (*f*_2, 17_ = 2, *p* = 0.17, *η*_*p*_^2^ = 0.19). Compared to the baseline measurement, total twitch torque was 5% (± 2%) (*p* < 0.01) and 7.7% (± 3%) (*p* = 0.03) lower 2HR post-exercise in the low- and high-load conditions, respectively (Fig. 2A). No differences were found when comparing total twitch torque between baseline and 48HR. Soreness levels after exercise were similar between conditions. There was a main effect of time (*f*_1, 16_ = 32, *p* < 0.01, *η*_*p*_^2^ = 0.67) on soreness scores, but no effect of load (*f*_1, 11_ = 0.9, *p* = 0.35, *η*_*p*_^2^ = 0.08) nor an interaction effect (*f*_2, 18_ = 2, *p* = 0.16, *η*_*p*_^2^ = 0.18). In both load conditions, the soreness scores increased at 2HR (high and low *p* < 0.01) and 48HR (high and low *p* < 0.01) after the exercise bout in comparison to baseline (Fig. 2B).

MG passive stiffness did not differ between load conditions, as we found a main effect of time (*f*_2, 20_ = 5, *p* = 0.02, *η*_*p*_^2^ = 0.33) but not effect of load (*f*_1, 11_ = 0.2, *p* = 0.66, *η*_*p*_^2^ = 0.02) nor an interaction effect (*f*_2, 17_ = 0.5, *p* = 0.54, *η*_*p*_^2^ = 0.06). Compared to baseline measurements, MG passive stiffness was higher 2HR (*p* = 0.04) and 48HR (*p* = 0.04) post-exercise in the low-load condition only (Fig. 2C).

There were no main effects or interaction effect for the measurement of active fascicle length at the maximum twitch torque (Fig. 2D).

**Fig. 3:**
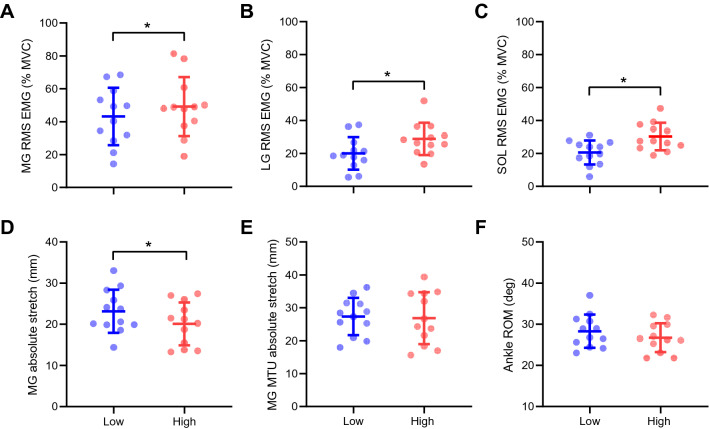
Medial gastrocnemius neuromechanical behaviour during heel drop bouts with low- and high-load levels. **A** medial gastrocnemius (MG) root mean square (RMS) activity as a percentage of the maximal voluntary isometric contraction (MVIC); **B** Lateral gastrocnemius (LG) RMS as a percentage of the MVIC; **C** Soleus RMS EMG as a percentage of the MVIC; **D** MG fascicle stretch amplitude; **E** MG muscle–tendon unit (MTU) stretch; **F** ankle range of motion (ROM). These variables were analysed during a bout of heel drops performed under low-load (body weight) or high-load (body weight + 30% body weight) conditions (*blue* and *red,* respectively). Individual values are presented as (circles), together with means and standard deviations (error bars). *Significant difference when comparing between force conditions (Low vs High) (*p* < 0.05)

Neuromechanical behaviour during the heel drops was different between load conditions. High-load caused higher triceps surae EMG activation (MG, *p* = 0.02, *η*_*p*_^2^ = 0.34; LG, *p* < 0.01, *η*_*p*_^2^ = 0.58; SOL, *p* < 0.01, *η*_*p*_^2^ = 0.48; Fig. 3A–C, respectively) and smaller (15%) MG absolute stretch (*p* = 0.02, *η*_*p*_^2^ = 0.39; Fig. 3D). However, MG MTU absolute stretch (*p* = 0.76, *η*_*p*_^2^ = 0.01; Fig. 3E) and ankle ROM (*p* = 0.25, *η*_*p*_^2^ = 0.11; Fig. 3F) did not differ between load conditions.

## Discussion

This study aimed to assess the effect of muscle force during stretch on EIMD in the MG muscle. The indirect markers of muscle damage indicated that EIMD was similar between low and high muscle load conditions. During the heel drop exercise, matched for total mechanical work performed, both load conditions resulted in similar MG MTU lengthening. However, the high-load condition had increased plantar flexor muscle activation resulting in less MG fascicle stretch. Overall, this study suggests that an increase in muscle load of 30% BW has no effect on the markers of muscle damage analysed when the amount of work absorbed over a series of contractions is controlled, likely because MG architectural features buffer muscle fibres against stretch.

Studies investigating the relationship between EIMD and muscle load (or force) during eccentric contractions in humans are scarce. Chen et al. (2007) demonstrated that in the elbow flexors, exercise intensity is related to the magnitude of EIMD, suggesting that muscle force magnitude is an important determinant of damage. In elbow flexors, increased damage during high-load eccentric contractions may reflect increased magnitudes of fascicle stretch (Lau et al. 2015). However, in this study, the high-load condition reduced the amount of fascicle stretch. Less active fascicle stretch was due to the role of the series elastic compliance of the muscle (Hoffman et al. 2014, 2016; Pincheira et al. 2018), which underwent most of the length change, even allowing muscle fibres to shorten slightly during the early stretch phase (Fig. 1). Architectural gearing in pennate muscles, whereby dynamic muscle shape change and fibre rotation during muscle stretch lead to velocities and magnitudes of stretch that are greater for the muscle belly than the fascicles (Azizi and Roberts 2014), may also play a buffering role. Further, the stretch occurs at short starting lengths, which has been also associated with less muscle damage (Talbot and Morgan 1998; Guilhem et al. 2016). Taken together, previous literature and our results suggest that the effects of force on fascicle stretch and thus muscle damage is likely to be specific for each muscle, due to different muscle architectures. Nevertheless, investigations assessing muscles with different architectures are necessary to fully understand the effect of muscle architecture on EIMD markers when high-low loads are used. Future studies should aim to explore this relationship in more detail.

Higher levels of EMG activity were found during the high-load condition, implying a greater number of actively stretched fibres and/or an increase in motor unit discharge rate during each cycle. While this may suggest an increase in the number of fibres susceptible to stretch-induced damage, factors such as fatigue may influence the population of fibres recruited to meet the demands of the task. For example, a recent study utilising high-density EMG found that a higher volume of muscle activity is needed to maintain a given force output after the cessation of a large number of eccentric cycles of the triceps surae muscles, compared to before the exercise (Pincheira et al. 2021b). It is conceivable that the high-load condition might induce greater fatigue than the low-load condition and hence there may be more requirement to distribute the force amongst a greater number of fibres (increased volume of active muscle). This response would effectively decrease the stress applied to each fibre, which may have acted to counteract the damage of fibres during the high-load contractions. Differences in muscle activation patterns between participants (Pincheira et al. 2021b) may help to explain the interindividual variability in the amount of muscle damage seen here and elsewhere (Guilhem et al. 2016).

On the other hand, non-intuitively, within a smaller volume of active muscle for the low-load condition, force would be high in some fibres (due to less redistribution), possibly predisposing them to greater damage. This assertion may be supported by the fact that passive stiffness was elevated after the exercise in the low-load condition only (Fig. 2C). However, the activation requirements (i.e., motor unit recruitment and rate coding) during each load task requires further consideration in determining how muscle activity influences muscle damage.

In this study, the total exercise volume for each condition (leg) was the same. In other words, the mechanical work in the low (BW * ~ 172 contractions) and high (BW + 30% BW * ~ 92 contractions) load conditions was the same, ~ 167 J/kg. Thus, any difference found between conditions is expected to be the result of the higher load in the BW + 30% BW condition rather than exercise volume. Our results show that there were no differences in damage metrics between load conditions, so it can be speculated that this was due to a reduced capacity of the high load to induce damage in the MG (given the decreased operating fascicle stretch). This finding is in partial agreement with hour hypothesis, as we expected lower damage in the high force load condition. However, it could also be possible that the damage elicited in the low force load condition was equivalent to that of the high force load because of the increased number of contraction cycles and the MG operating with a larger absolute stretch magnitude. Nevertheless, even if the latter is the case, our results still suggest that high loads (BW + 30% BW) do not elicit much damage in the MG muscle. It is also worth noting that several previous studies (Hoffman et al. 2016; Guilhem et al. 2016; Pincheira et al. 2018, 2021b), using different tasks and loads, have failed to elicit high levels of muscle damage in the gastrocnemius muscle (e.g., force drops larger than ~ 20% MVIC). Thus, it seems that in this muscle, in humans, in vivo, high levels of EIMD are not achievable due to the damage-buffering characteristics mentioned above and elsewhere (Butterfield 2010).

The results of this study should be interpreted considering some limitations. Defining exercise loads relative to MVICs may also be desirable and may lead to different results (given the different standardization of the exercise load). However, the % of BW as employed in the current study, has been used successfully previously to standardise training/exercise loads for lower limb muscles (Alfredson et al. 1998; Mafi et al. 2001; Pincheira et al. 2021a). It is possible that the increase in 30% BW may not have been enough to elicit the response/adaptations hypothesised in the markers of muscle damage analysed. However, as mentioned before, higher loads would have made it difficult to perform repetitive eccentric contractions while controlling the kinematics of the task and thus MTU stretch. Further studies are necessary to investigate the impact of higher loads on both the markers of muscle damage utilised in this study and other potential markers (e.g., inflammatory) in ecologically valid eccentric exercise interventions. Our results show that in the high-load group, ~ 92 contractions were needed to match the mechanical work of the low-load condition. This represents an ~ 45% decrease in the number of cycles where we would have expected a decrease in 30%. This discrepancy may be because of subtle differences of where the added mass was positioned (i.e., location of the weight-vest in relationship to the centre of mass of the participant) and how this corresponds to work done per cycle; or due to slightly different movement strategies during the heel drops in each load condition (e.g., a slight lift of the centre of mass coming from the knee in one condition vs. the other). Further studies may be needed to elucidate the effect of movement kinetics/kinematics on mechanical work during ecologically valid exercise protocols. We only assessed architectural changes in MG, whereas damage likely occurred in all muscles of the triceps surae. As such, we are unable to conclude whether the finding of decreased fascicle stretch is ubiquitous across all triceps surae muscles. Since both exercises occurred two weeks apart, a contralateral repeated bout effect may have occurred between legs. However, given that the load condition (low or high) occurring on the first session was randomised, and that the magnitude of the repeated bout effect is smaller in the gastrocnemius compared to other muscles (Hoffman et al. 2016; Pincheira et al. 2018, 2021b), we suspect that any contralateral repeated bout effect would be small.

## Conclusion

Increasing muscle load from BW to BW + 30% BW during eccentric contractions has only a modest impact on MG EIMD, at least when the total mechanical work performed is controlled. Based upon previous literature, we speculate that the absence of a pronounced effect of load on damage may be due to muscle architectural features (i.e., high degree of fibre pennation and series elastic compliance) or neural mechanisms (i.e., higher muscle activation distributing workload among a larger number of muscle fibres) that effectively buffer muscle fibres from damage under high-loading forces. Future studies should consider assessing whether at matched loads, increasing the number of eccentric stretch cycles increases the likelihood of MG damage.

## Data Availability

Data will be available upon reasonable request.

## Abbreviations

ANOVA: Analysis of variance
BW: Body weight
EIMD: Exercise-induced muscle damage
EMG: Electromyography
LG: Lateral gastrocnemius
MG: Medial gastrocnemius
MTU: Muscle–tendon unit
MVIC: Maximal voluntary isometric contraction
PRE: Before the exercise bout
RMS: Root mean square
ROM: Range of motion
SOL: Soleus
2H: Two hours
48H: Forty-eight hours
